# Mutation analysis of the *ATR *gene in breast and ovarian cancer families

**DOI:** 10.1186/bcr1037

**Published:** 2005-05-06

**Authors:** Katri Heikkinen, Virpi Mansikka, Sanna-Maria Karppinen, Katrin Rapakko, Robert Winqvist

**Affiliations:** 1 Department of Clinical Genetics, Oulu University Hospital/University of Oulu, Oulu, Finland

## Abstract

**Introduction:**

Mutations in *BRCA1*, *BRCA2*, *ATM*, *TP53*, *CHK2 *and *PTEN *account for only 20–30% of the familial aggregation of breast cancer, which suggests the involvement of additional susceptibility genes. The ATR (ataxia-telangiectasia- and Rad3-related) kinase is essential for the maintenance of genomic integrity. It functions both in parallel and cooperatively with ATM, but whereas ATM is primarily activated by DNA double-strand breaks induced by ionizing radiation, ATR has been shown to respond to a much broader range of DNA damage. Upon activation, ATR phosphorylates several important tumor suppressors, including p53, BRCA1 and CHK1. Based on its central function in the DNA damage response, *ATR *is a plausible candidate gene for susceptibility to cancer.

**Methods:**

We screened the entire coding region of the *ATR *gene for mutations in affected index cases from 126 Finnish families with breast and/or ovarian cancer, 75 of which were classified as high-risk and 51 as moderate-risk families, by using conformation sensitive gel electrophoresis and direct sequencing.

**Results:**

A large number of novel sequence variants were identified, four of which – Glu254Gly, Ser1142Gly, IVS24-48G>A and IVS26+15C>T – were absent from the tested control individuals (*n *= 300). However, the segregation of these mutations with the cancer phenotype could not be confirmed, partly because of the lack of suitable DNA samples.

**Conclusion:**

The present study does not support a major role for *ATR *mutations in hereditary susceptibility to breast and ovarian cancer.

## Introduction

Of all breast and ovarian cancers, 5–10% are due to genetic predisposition [1]. Mutations in the two high penetrance genes *BRCA1 *and *BRCA2 *are well known, but they account for only 20–30% of the familial aggregation of breast cancer. The remaining cases could be the result of a few additional, yet unidentified, high penetrance mutations, but the polygenic model may provide a more plausible explanation [2]. According to this model, genetic susceptibility to breast cancer is due to several loci, each conferring a modest independent risk [3]. Because the protein products of the genes thus far associated with breast and/or ovarian cancer predisposition are central players in the pathways involved in cell cycle checkpoint functions, and in the sensing, transduction and repair of DNA lesions [4], other similarly acting genes may represent new potential candidates.

The ATR (ataxia-telangiectasia- and Rad3-related) kinase is essential for the maintenance of genomic integrity. It is a key activator of the cellular responses to DNA lesions [5]. In response to DNA double-strand breaks induced by ionizing radiation ATR, along with ATR-interacting protein, acts in parallel with ATM (ataxia-telangiectasia mutated), which is defective in the neurodegenerative disorder ataxia-telangiectasia and is also associated with breast cancer susceptibility [6-9]. Whereas ATM is responsible for the immediate and rapid response to double-strand breaks, ATR joins in later and maintains the phosphorylated state of specific substrates. However, this is not the main role played by ATR; it also responds to ultraviolet-induced lesions, stalled replication forks and hypoxia [5]. In response to these events, ATR phosphorylates key proteins in various branches of the DNA damage response pathways, such as p53, BRCA1, CHK1 and Rad17, thereby activating DNA repair, cell cycle checkpoints, or apoptosis [10,11].

The cellular functions of ATR are indispensable, as demonstrated in mice, in which biallelic disruption of *ATR *leads to early embryonic lethality. In contrast, *ATR*^+/- ^mice exhibit only a small decrease in survival but tumor incidence is increased [12]. In humans a connection between *ATR *defects and tumorigenesis has also been suggested, mainly by studies reporting somatic changes in *ATR *in gastric and endometrial cancers exhibiting microsatellite instability [13,14]. Consequently, it has been proposed that *ATR *serves as a haploinsufficient tumour suppressor on a mismatch repair deficient background [15].

Recently, inherited defects in ATR signalling were shown to associate with Seckel syndrome, because patients in two families were found to be homozygous for a hypomorphic *ATR *mutation. Seckel syndrome is a heterogenous recessive disorder that is characterized by dwarfism, developmental delay and severe microcephaly. It shares an overlap in clinical features with two recessive cancer susceptibility syndromes, Nijmegen breakage syndrome and Fanconi anemia (FA) [16,17]. Interestingly, the gene that is defective in two FA complementation groups, namely FA-B and FA-D1, has been identified as *BRCA2 *– a major breast cancer susceptibility gene [18,19]. In addition, carriers of the Nijmegen breakage syndrome Slavic founder mutation appear to be at increased risk for breast cancer [20]. Thus far, predisposition to cancer has not been reported in patients with Seckel syndrome. However, various cell lines in which *ATR *has been inactivated exhibit genetic instability, and this may predict proneness to cancer [17].

Based on this, we wanted to determine whether *ATR *germline mutations are involved in susceptibility to breast and/or ovarian cancer, and conducted a mutation analysis of all 47 coding exons and exon–intron boundaries in the affected index cases in 126 families.

## Materials and methods

### Subjects

The index cases of 126 breast and/or ovarian cancer families originating from northern Finland were screened for *ATR *germline mutations. Of the studied families, 94 were affected by breast, 29 by breast/ovarian and three by ovarian cancer. All index cases had been diagnosed with either breast or ovarian cancer. 75 of the families were classified as high-risk families and were defined as follows: three or more cases of breast and/or ovarian cancer in first- or second-degree relatives; or two cases of breast and/or ovarian cancer in first- or second-degree relatives, of which at least one had early disease onset (age ≤35 years), bilateral disease, or multiple primary tumours. Most of the high-risk families contained three or more cancer cases. The remaining 51 families contained two cases of breast and/or ovarian cancer in first- or second-degree relatives and were considered to be at moderate disease risk. All of the high-risk families had previously been screened for germline mutations in *BRCA1*, *BRCA2*, *CHK2 *and *TP53 *[21-23] and 10 families were known to have disease-related mutations in *BRCA1 *or *BRCA2*. The frequencies of all observed germline variants were assessed in either 100 or 300 control individuals, who were anonymous cancer-free blood donors originating from the same geographical region as the studied families.

All patients gave informed consent for acquisition of pedigree data and blood specimens for use in a study on cancer susceptibility gene mutations. Approval to perform the study was obtained from the Ethical Board of the Northern Ostrobothnia Health Care District and the Finnish Ministry of Social Affairs and Health.

### Mutation analysis

DNA was extracted from blood lymphocytes using either the standard phenol–chloroform protocol or the Puregene D-50K purification kit (Gentra, Minneapolis, MN, USA). Screening of the protein encoding and exon–intron boundary regions of *ATR *was done by conformation sensitive gel electrophoresis (CSGE), which is a cost-efficient way to scan for mutations with high detection sensitivity and specificity [24,25], or by direct sequencing. Sequencing analysis was performed using the Li-Cor IR^2 ^4200-S DNA Analysis system (Li-Cor Inc., Lincoln, NE, USA) and the SequiTherm EXCEL™II DNA Sequencing Kit-LC (Epicentre Technologies, Madison, WI, USA). Oligonucleotides for CSGE and sequencing (Table 1) were designed using Primer3 software [26], utilizing sequence information obtained from public databases. Polymerase chain reaction conditions for CSGE and sequencing are available upon request.

### Statistical analyses

Fisher's exact test or χ^2 ^test was used to determine statistical significance (SPSS version 12.0 for Windows; SPSS Inc., Chicago, IL, USA). All *P *values were two sided.

## Results

**Figure 1:**
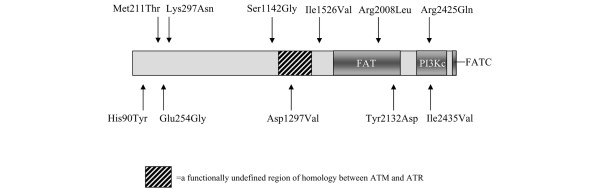
Structure of ATR and the location of observed amino acid changes.

Mutation analysis revealed several alterations in the *ATR *gene. Altogether, 23 nucleotide substitutions were observed: 17 in the exon and six in the intron regions (Tables 2 and 3). Eleven of the exonic changes resulted in amino acid substitutions, eight of which were novel and three were polymorphisms reported in the single nucleotide polymorphism database [27]. The location of the amino acid changes is summarized in Fig. 1. All observed nucleotide alterations were assessed for possible effects on splicing consensus sequences [28], and the coding sequence variants were tested using the ESEfinder program [29] to identify those that reduced the exonic splicing enhancer (ESE) score below the calculated threshold.

Of the observed amino acid substitutions, four were located in known functional sites: Arg2008Leu and Tyr2132Asp to the FAT (FRAP/ATM/TRRAP) domain, and Arg2425Gln and Ile2435Val to the PI3Kc (phosphoinositide 3-kinase related catalytic) domain. The novel Arg2008Leu, Tyr2132Asp and Ile2435Val alterations occurred in one index case each. Interestingly, Arg2008Leu appeared to have an effect on two ESEs (SF2/ASF and SC35), as predicted by the ESEfinder program.

Arg2008Leu was identified in a patient with both ovarian and colon cancer at age 51 years, and her sister, diagnosed with breast cancer at age 72 years, was found to be a carrier. However, because Arg2008Leu, Tyr2132Asp or Ile2435Val carriers were also observed in control individuals, these changes were all classified as rare variants. Arg2425Gln is a common polymorphism described in the single nucleotide polymorphism database, and its frequency was similar in cases (27.8%) and controls (24.0%).

The rest of the amino acid substitutions were all located outside the kinase and FAT/FATC domains, although two of these, Glu254Gly and Ser1142Gly, were absent from the tested controls. Glu254Gly affects a nonconserved residue, and was seen in one patient diagnosed with breast cancer at age 37 years. However, her maternal cousin, with bilateral breast cancer at ages 45 and 55 years, was not a carrier. The other change, Ser1142Gly, affects a residue that is also conserved in ATR of *Xenopus laevis *and in mei41 of *Drosophila melanogaster *[30]. Ser1142Gly was seen in two index cases with breast cancer (2/126; *P *= 0.09). In the first family the patient was diagnosed at age 64 years. Two of her daughters had breast cancer at ages 49 and 40 years, but both tested negative for Ser1142Gly. Also, two sisters of the index had breast cancer at unknown ages, but no samples were available for mutation testing. In the second family the index patient was diagnosed at age 59 years, but her sister, who had breast cancer at age 45 years, was not a carrier. Neither Glu254Gly nor Ser1142Gly had an affect on splicing consensus sequences or ESEs.

Of the six intronic changes, two were absent from the tested control individuals: IVS24-48G>A was observed in the index case of three families (3/126; *P *= 0.03) and IVS26+15C>T was observed in one case. Unfortunately, only one additional DNA sample from an affected relative was available for mutation testing, and this maternal cousin of the index case proved negative for IVS24-48G>A. Also IVS31-74G>A was found more frequently in cases (8.7%) than in control individuals (4.3%), but the difference was only marginally significant (odds ratio 2.1, 95% confidence interval 0.92–4.85; *P *= 0.07). None of the observed intron changes had an affect on consensus splice sites.

## Discussion

ATR plays a critical role in the maintenance of genomic integrity. A number of tumour suppressor proteins act downstream of ATR, placing it high in the DNA damage response cascade [5]. Impaired ATR signalling has been shown to result in Seckel syndrome, but thus far predisposition to cancer in these patients has not been reported [16]. Nevertheless, cell lines with inactivated *ATR *exhibit genetic instability, which may suggest proneness to cancer [17].

To investigate the possible role played by *ATR *germline mutations in hereditary predisposition to breast and ovarian cancer, the whole coding region of the gene was screened for mutations in the index cases from 126 families. We found a number of novel sequence variants, but we did not identify any clearly pathogenic alterations. Only two of the observed missense changes, Glu254Gly and Ser1142Gly, were absent from control individuals. However, because the variants did not segregate with the cancer phenotype in these families, they are unlikely to be important cancer susceptibility alleles. Evaluation of the intronic variants IVS24-48G>A and IVS26+15C>T is more difficult because only one additional DNA sample was available for mutation testing, but neither had any affect on consensus splicing sequences. The possible association of the identified rare variants with predisposition to cancer must be demonstrated by more extensive case–control studies.

The performed mutation analysis is to our knowledge the first to investigate the possible association of germline *ATR *mutations with cancer predisposition. However, the results of the study suggest that *ATR *is not involved in hereditary susceptibility to breast and ovarian cancer. The lack of deleterious germline mutations could reflect a fundamental role for ATR in cell viability, including DNA replication [5]. Nonetheless, because *ATR *changes have thus far been reported only in gastric and endometrial tumours exhibiting microsatellite instability, it is also possible that breast and/or ovarian cancer is not the primary cancer phenotype associated with germline mutations in this gene [13,14]. These findings need confirmation by other studies.

## Conclusion

Based on its central role in the maintenance of genomic integrity, we hypothesized that germline mutations in *ATR *may account for some breast and/or ovarian cancer families. However, analysis of 126 index cases suggests that *ATR *mutations do not play a major role in hereditary susceptibility to these cancers.

## Abbreviations

ATM= ataxia-telangiectasia mutated; ATR = ataxia-telangiectasia- and Rad3-related; CSGE = conformation sensitive gel electrophoresis; ESE = exonic splicing enhancer; FA = Fanconi anemia.

## Competing interests

The author(s) declare that they have no competing interests.

## Authors' contributions

KH and VM conducted the screening studies. RW, KH and VM participated in the design of the study and performed the statistical analysis. S-MK and KR helped to draft the manuscript. All authors read and approved the final manuscript.

## Figures and Tables

**Table 1:** Primers used to amplify exons and splice junctions of *ATR*

Exon	Forward^a^	Reverse
1	ccgggtcctatgcagaaaag	aggggagagcacgtgaaac
2	cattgacactgaacacatttgatg	tctaaaactacatggagaaaatgct
3	ggcccacagtctggtttct	gtaatatttcagaagagcagtaaaagg
4a	tcgtcaaggatttagcaaatga	acgagtaagaaccattaataaagtgac
4b	atgtgatgggtcatgctgtg	gctcttcatagagtttcaattggtc
4c	tccaaaaattaaatccctagcaa	tctcacatagaccttcctgacttg
4d	aaactctgtgtcatgtttgaagac	gccagactacactatgaaaatcatta
5	cattcttgctgcctatgaataa	aaatcaaagcacttaactaaagctga
6	tctaaatatgtttcatgttttaaccaa	tgagtcaagtgaataatgagtaaaca
7	tggctttactacaattttatgtttgac	cacttaggcttcaggcaaaa
8^b^	tttaaaagagatatgattaaggaaaag	cacacattcttgtgagcactt
9	aaatgtattttaagtgttacttgactttt	aaccctgcatacatagccaga
10	gtcccaaattaagcaagactattt	caaggcttcagtctaattcttttac
11	tcatggcatattatttgttgac	gaacaataaaattaactggttaaagaa
12	gaggttgataatttttgtttttaacat	ccatttttaacagcaagcaaa
13	tgagtcaacatgaatttatttgtagat	aaagaaaagcaagcaaaataaaac
14	ctctatggtggcttaaaaagtattagt	caaagtcaaaatctagaatggaatg
15	ctccaaatatgtgtggcattt	accctctttcctagaagaatgttac
16	ctcctgatgtactaatagcatgttaaa	tgaccaaaaatatgatttcttcaat
17	gcttttggagaaacttaattaacca	tgtttgtagctagatgcagaattt
18^b^	tgtccttagggctcatctgc	tgaacccaatttccctcaaa
19	gctgccttttaatctattgtttg	cattaccatcagtaattttgagacat
20	ggccttagtttcaacttttactttaca	caggaattagctatcagaataggact
21	gagaattcaggcctttggaa	aatgtcattttgtcatcttttcttt
22	aactcatcaaaaactagctgaaaaa	ggataagctgaatagttctttgtaaat
23	ccatggaaaaagcagtacacc	aaaacaaaaaggagtttcacaagt
24	gcataaataaagcgaagtgcaa	ggccaaaaaaatcgcatta
25	agtcaactgaaggagttgctg	ttgtgtgtgctaggcattcag
26	ttatctcacatgctactctttgaca	catttcctactaataggtagcctttc
27	ttagaatggttagctttagatgtcata	agaactgataaagggaagagctaa
28	caattgttctgttgttagttacattct	gcatagcatataaaacattcaataaaa
29	aaacaggtggttttatagttttatttc	aaggtttccagagttcctattca
30	aagggcaataaggtaaatagtaat	aaattacccaattcactaactaaaaa
31	gaacaaaatacaatataatgcaaattcaa	accgcacccatcctaaaact
32	ttgatatttcagctgaccattttatc	ccaaactcactatcaattatttactcaaa
33	caactgtgtattttaaattctttatttctg	cacccccaaataatatccaa
34^b^	attgggaacagaggctttca	gacatttccctggccattac
35	caaaaacataatgaactaatacttttgc	catgtgctttgccatatagactt
36	tcacatacttttgatccctaatca	acctagaatatgctaagacatgtga
37	tttttgtgaaaacggtatgtgg	agactgtccagccaaatctga
38	tgtgaaatgaactgatatactgattttt	cgccctggaacttgtatcta
39	aactctcatcatgaatactttttaagtt	aaaaactgctttattaagacaaatcat
40	ttgtaaaagtgaaatttttgttatagtgg	ttgtgaaatacactttttatcttaatttga
41	tttacacagaaatttttggcccta	caactctgaaataaaagcaatctgg
42	tttggttatgaaatgaacaatcttt	aggaagggatggaaacactt
43	agtagatgtttcttgtccaattttaac	catatgaggccaatataaatctaaaa
44	gttgttatggttgaatgtttattttta	caaggaagatacagttgttgagaa
45	tggacatgaagttctttgagtaaa	caaacatatgtaggggccaat
46	agcttctcatccttcacttaaa	aactatagctgcatatcaagttca
47a	gggtattggtcagtaaaatggta	ccacagattcataccaaatgc
47b	gaaggacatgtgcattaccttatac	cttgcttgtttcttgcaaatatag

^a^All primer sequences are shown in the 5' to 3' direction. ^b^The amplified fragment was analyzed by direct sequencing.

**Table 2:** Observed sequence variation in the protein-encoding regions of *ATR*

Exon	Nucleotide change	Effect on protein	Carrier frequency		*P*	Status^a^
					
			Familial cases	Controls		
3	268C>T	His90Tyr	12.7% (16/126)	10.3% (31/300)	0.50	Novel
4	632T>C	Met211Thr	54.0% (68/126)	47.3% (142/300)	0.20	Reported
	761A>G	Glu254Gly	0.8% (1/126)	- (0/300)	0.30	Novel
	891G>C	Lys297Asn	1.6% (2/126)	2.3% (7/300)	1.00	Reported
						
8	1776T>A	Gly592Gly	50.8% (64/126)	41.0% (41/100)	0.18	Reported
	1815T>C	Asp605Asp	46.8% (59/126)	49.0% (49/100)	0.79	Reported
14	2946C>T	Phe982Phe	0.8% (1/126)	2.0% (6/300)	0.68	Novel
17	3424A>G	Ser1142Gly	1.6% (2/126)	- (0/300)	0.09	Novel
21	3893A>T	Asp1297Val	0.8% (1/126)	1.0% (3/300)	1.00	Novel
26	4576A>G	Ile1526Val	4.0% (5/126)	2.0% (6/300)	0.31	Novel
30	5208T>C	Tyr1736Tyr	23.8% (30/126)	37.0% (37/100)	0.03	Reported
32	5459T>C	Tyr1820Tyr	26.2% (33/126)	31.0% (31/100)	0.43	Reported
35	6023G>T	Arg2008Leu	0.8% (1/ 126)	0.3% (1/300)	0.51	Novel
38	6394T>G	Tyr2132Asp	0.8% (1/126)	0.3% (1/300)	0.51	Novel
43	7274G>A	Arg2425Gln	27.8% (35/126)	24.0% (72/300)	0.46	Reported
	7303A>G	Ile2435Val	0.8% (1/126)	0.3% (1/300)	0.51	Novel
47	7875A>G	Gln2625Gln	27.0% (34/126)	35.0% (35/100)	0.19	Reported

^a^Novel or reported in the National Center for Biotechnology Information single nucleotide polymorphism database .

**Table 3:** Observed sequence variation in the intron regions of *ATR*

Location	Nucleotide change	Carrier frequency		*P*	Status^a^
				
		Familial cases	Controls		
IVS2-51	A>T	9.5% (12/126)	9.0% (9/100)	0.89	Novel
IVS16-26	T>A	2.4% (3/126)	4.3% (13/300)	0.41	Novel
IVS18-22	G>C	4.8 % (6/126)	4.0% (12/300)	0.72	Novel
IVS24-48	G>A	2.4% (3/126)	- (0/300)	0.03	Novel
IVS26+15	C>T	0.8% (1 /126)	- (0/300)	0.30	Novel
IVS31-74	G>A	8.7% (11/126)	4.3% (13/300)	0.07	Novel

^a^Novel or reported in the National Center for Biotechnology Information single nucleotide polymorphism database .
